# BMI1s interact with condensin complexes to regulate chromatin 3D structure and gene expression in *Arabidopsis*

**DOI:** 10.1007/s42994-025-00202-x

**Published:** 2025-02-17

**Authors:** Lingxiao Luo, Minqi Yang, Yue Zhou

**Affiliations:** State Key Laboratory of Gene Function and Modulation Research, School of Advanced Agricultural Sciences, Peking-Tsinghua Center for Life Sciences, Peking University, Beijing, 100871 China

**Keywords:** Chromatin 3D structure, Compartment domains, BMI1s, Condensin, Co-regulation

## Abstract

The accurate chromatin states are essential for maintaining genome integrity and ensuring the normal transcription of genes. Polycomb group (PcG) proteins regulate chromatin states not only by modifying the chromatin, but also by influencing the chromatin three-dimensional (3D) structure. The core components of Polycomb repressive complex 1 (PRC1), B LYMPHOMA MOLONEY MURINE LEUKEMIA VIRUS INSERTION REGION 1 HOMOLOG 1A/B/C (BMI1s), have been reported to maintain the compartment domains (CDs) generally, but the mechanism by which they function remains elusive. Here, we reveal that condensin complexes, whose function are related to chromatin or chromosome, can interact with BMI1s. Removal of condensin I or II also leads to global impairment of CDs. The significantly impaired CDs in *bmi1a/b/c* and condensin mutants are basically the same and the CDs co-regulated by BMI1s and condensin complexes have higher strength in the wild-type (WT, Col-0) plant, indicating that BMI1s and condensin complexes cooperate to maintain CDs. This regulatory function is parallel to the function of histone modifications deposited by PcG in maintaining CDs, since removal of either condensin I or II does not obviously disrupt the genome-wide level of H3K27me3 and H2AK121ub. Moreover, we discovered that BMI1s and condensin complexes jointly influence the expression of a portion of genes to enable normal plant growth and may maintain the genome integrity under stress conditions. Thus, our work proides a perspective for the gene expression and epigenetic regulatory mechanism of PRC1, in *Arabidopsis*, in addition to histone modifications.

**Supplementary Information:**

The online version contains supplementary material available at 10.1007/s42994-025-00202-x.

## Introduction

The accurate chromatin states are essential for maintaining the genome integrity and ensuring the normal transcription of genes (Bourbousse et al. 2019; Zheng and Xie 2019). In interphase, the chromatin should be folded orderly in a nucleus of a certain size, which is defined as the chromatin three-dimensional (3D) structure. A growing body of research shows that the chromatin 3D structure generally includes territories, compartments, topologically associated domains (TADs), and chromatin loops at different scales (Dixon et al. 2012; Feng et al. 2014; Grob et al. 2014; Lieberman-Aiden et al. 2009; Sexton et al. 2012). The genome can be segmented by highly self-interacting domains, which are widely referred to as TADs. Such domains are slightly different among different species and according to the characteristics, they can be called other names. In mammalian cells, TADs usually refer to the structures formed by the CCCTC-binding factor (CTCF) and cohesin complex (Dixon et al. 2012; Fudenberg et al. 2016; Sanborn et al. 2015; Szabo et al. 2019; Zheng and Xie 2019). However, TADs in Drosophila are well correlated with chromatin states and can be divided into transcriptionally active-TADs, heterochromatin-TADs, H3K27me3-TADs, and null-TADs (Ramírez et al. 2018; Sexton et al. 2012). Besides, dCTCF is one but not the only kind of architectural proteins binding to TAD boundaries, and not the key for regulating TAD structure (Ramírez et al. 2018; Van Bortle et al. 2014; Wang et al. 2018). In plants, the situation is more similar to that in *Drosophila* (Dong et al. 2018, 2020; Grob et al. 2014; Ouyang et al. 2020; Rowley et al. 2017; Yin et al. 2023). The compacted domains can be observed in the Hi-C matrix and they are also overlapped with and can be classified by different histone modifications, exhibiting a well correspondence with the compartment. Therefore, TADs in *Drosophila* and *Arabidopsis* are also referred to as the TAD-like or compartment domains (CDs). Recently, three types of histone modification dominated CDs, which are H3K4me3-, H3K27me1- (or H3K9me2-), and H3K27me3-CDs, and null-CDs without dominated histone modification are identified in *Arabidopsis* (Yin et al. 2023). However, the formation and regulatory mechanisms of CDs have always been a key and unresolved issue in plant epigenetics.

The tight relationship between histone modifications and chromatin 3D structures has been widely recognized and studied (An et al. 2024; Feng et al. 2014; Huang et al. 2021). As for CDs in plants, it has been reported that H3K27me3 and H2AK121ub are important to maintain intra-CD interactions (Yin et al. 2023). Except for histone modifications, other 3D regulators, especially functional proteins like architectural proteins in Drosophila are also consistently searched for. Initially, plant-specific Teosinte-branched 1/Cycloidea/Proliferating (TCP) transcription factors have been reported to be enriched at TAD boundaries in Marchantia. The DNA motif recognized by TCPs is also significantly enriched in the TAD/CD boundaries in rice, maize, and *Arabidopsis* (Karaaslan et al. 2020; Liu et al. 2017; Sun et al. 2020; Yin et al. 2023). However, the correlating domain structure is not impaired by mutating *TCPs* in Marchantia, indicating that the organizing function of TCPs still needs to be confirmed and the boundary regulation still needs to be studied (Doğan and Liu 2018; Liu et al. 2017). Recently, plant-specific PWWP-DOMAIN INTERACTOR OF POLYCOMBS (PWO) proteins have been discovered to specifically maintain the H3K27me3-CDs, including the boundary strength, the interactions within H3K27me3-CDs and the repressive chromatin states, which is parallel to H3K27me3/PcG function (Yang et al. 2024). EMBRYONIC FLOWER1 (EMF1), as a PcG protein unique to plants, has been reported to maintain the boundary strength of all kinds of histone modification-dominated CDs, either independently or in cooperation with histone modifications (Shu et al. 2024). Therefore, a growing body of evidence shows that multiple functional proteins, which directly or indirectly regulate chromatin 3D structure, are also essential in plants. Such proteins still need to be identified, and the mechanisms underlying the formation, or maintenance, of CDs needs to be explored.

PcG proteins, first identified in *Drosophila*, modify the chromatin to accurately control the spatiotemporal expression of genes (Lewis 1978; Mozgova and Hennig 2015). PcG proteins form two distinct complexes, PRC1 and 2. PRC1 owns H2A ubiquitin ligase activity, whereas PRC2 has H3K27 tri-methyltransferase activity (Bratzel et al. 2010; Cao et al. 2002; Czermin et al. 2002; Müller et al. 2002; Pien and Grossniklaus 2007; Wang et al. 2004). In addition to mediating the deposition of histone modifications, PcG proteins have been reported to be involved in chromatin 3D regulation in different species, such as human (Cai et al. 2021), mouse (Blackledge et al. 2014; Gentile et al. 2019), and flies (Bantignies et al. 2011; Loubiere et al. 2020). Similar to mammals and *Drosophila*, the combined activity of PRC1 and PRC2 is required to maintain the interactions within H3K27me3-CDs in *Arabidopsis*. However, the distributions of H3K27me3 and H2AK121ub in *Arabidopsis* do not overlap so well in comparison to mammalian cells, and H2AK121ub modifications are irregularly distributed in all four types of CDs (Yin et al. 2023). In addition, when losing the PRC1 ubiquitin ligase BMI1s, the CDs are impaired globally rather than H3K27me3-CDs only. Therefore, the mechanisms of BMI1s regulating the general CD structure remain unclear and the phenomenon suggested that PRC1 may have other 3D regulatory mechanisms independent of PRC2 activity or its ubiquitin ligase activity.

The condensin complexes, which belong to the structural maintenance of chromosome (SMC) complex, were first identified in *Xenopus* and exist in both prokaryotes and eukaryotes (Hirano et al. 1997; Kimura and Hirano 1997; Ono et al. 2003; van Ruiten and Rowland 2018). Most eukaryotes have two types of condensin complexes, condensin I and condensin II, both of which consist of two core SMC proteins (SMC2 and SMC4), one kleisin superfamily protein (Chromosome-Associated Protein H/H2, CAP-H/H2) and two HEAT repeat domain proteins (CAP-D2/3 and CAP-G/G2). The two complexes share the core SMC components (SMC2 and SMC4) but have distinct non-SMC proteins (CAP-D2, CAP-G, CAP-H belong to condensin I and CAP-D3, CAP-G2, CAP-H2 belong to condensin II) (Kalitsis et al. 2017; Rana and Bosco 2017). Condensin complexes are well known for condensing the loose chromatin to the short and thick chromosomes to enable the normal mitotic or meiotic process (Batty and Gerlich 2019; Kalitsis et al. 2017; Kimura and Hirano 1997; Yanagida 2009). Condensin complexes were also detected to function in interphase, such as participating in chromatin 3D regulation (Hagstrom and Meyer 2003; Kim 2021; Yuen and Gerton 2018). For example, condensin II have been reported to function in determining the architecture type in eukaryotes or the formation of A compartment in mammalian cells (Bauer et al. 2012; Hoencamp et al. 2021; Yuen et al. 2017). In *Arabidopsis*, condensin II can prevent abnormal centromere association and maintain the association between centromeres and rDNA arrays (Sakamoto et al. 2019; Wallace and Bosco 2013). Recently, condensin II was reported to incorporate with the linker of the nucleoskeleton and cytoskeleton (LINC) complex to scatter the centromeres (Sakamoto et al. 2022). However, compared to the in-depth study of condensin function during cell division, there is limited research on the role of condensin in interphase, especially in plants. Therefore, the role of condensin in interphase deserves further exploration.

In this study, we identified a BMI1A interactor CAP-H, which is the subunit of condensin complexes, through yeast two-hybrid screening. We further confirmed the interactions between condensin I/II and BMI1s. However, removal of either condensin I or condensin II does not lead to the disruption of histone modifications deposited by PcG, indicating that BMI1s and condensin may cooperatively regulate other aspects. Through Hi-C experiments and detailed data analysis, we discovered that both condensin I and condensin II indeed generally influence the chromatin 3D structure. Although BMI1s, condensin I and condensin II have their individual roles in chromatin 3D regulation, they function together to maintain CDs. Moreover, the maintenance of the CDs that have strong interaction strength needs the cooperative roles of the three complexes. BMI1s and condensin complexes also co-regulate the expression of a portion of genes to enable normal plant growth and may maintain stability of the 3D genome under stress conditions. Our results clarified the role of condensin complexes in *Arabidopsis* to regulate CDs in concert with PRC1 from a perspective other than modifications, which supplements the chromatin 3D regulatory mechanisms of PRC1 and condensin complexes.

## Results

### BMI1s interact with condensin subunits CAP-H and H2

**Fig. 1:**
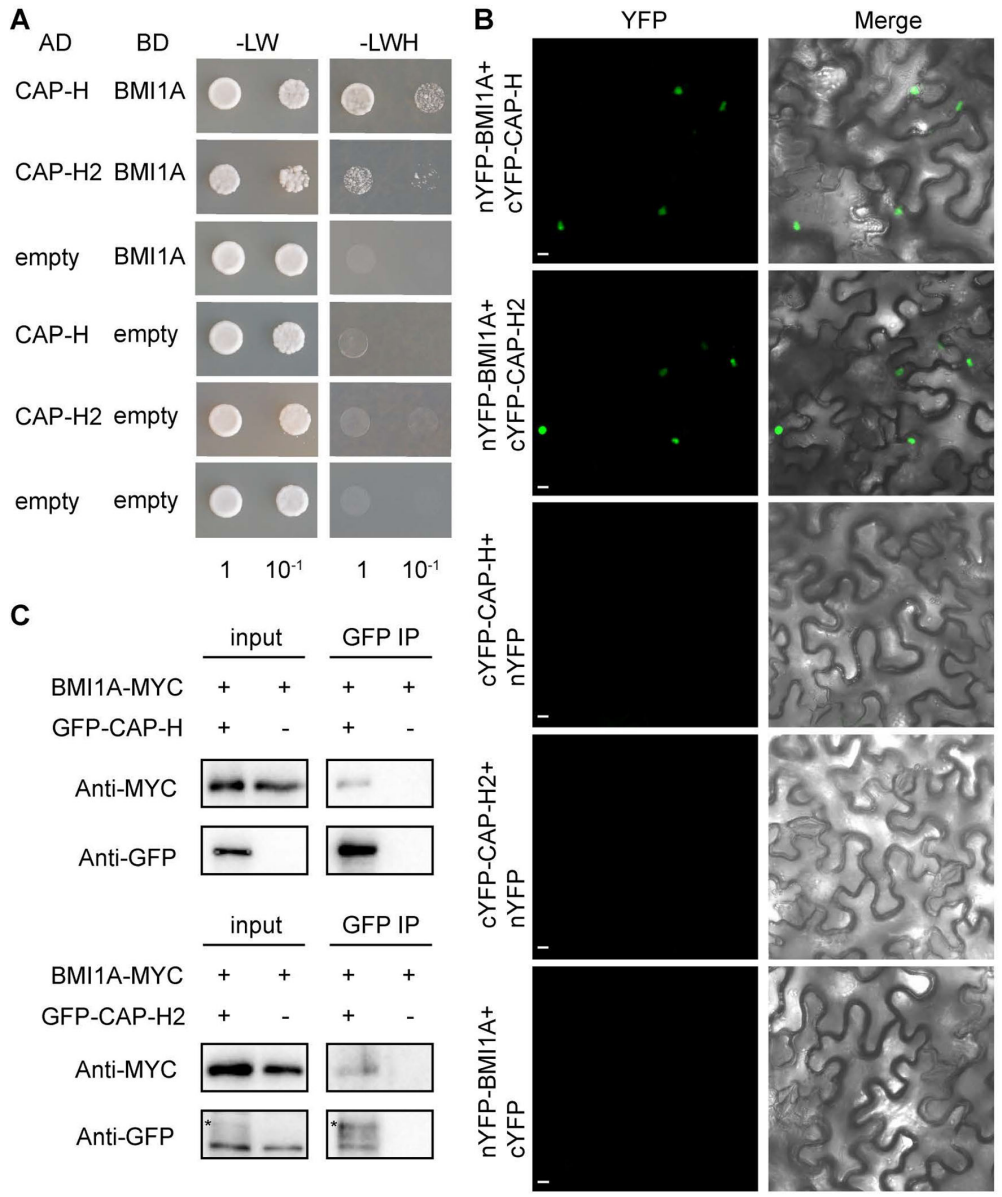
BMI1s interact with condensin subunits CAP-H and H2. **A** Yeast two-hybrid assays show that BMI1A interacts with CAP-H and CAP-H2. **B** The confocal images of BiFC assay in *Nicotiana benthamiana* leaf show the interaction between cYFP-CAP-H or cYFP-CAP-H2 and nYFP-BMI1A. nYFP-BMI1A represents the BMI1A fused with the N-terminal of YFP and cYFP-CAP-H/H2 represents the CAP-H/H2 fused with the C-terminal of YFP. Scale bar: 10 μm. **C** Co-IP experiments show the interaction between GFP-CAP-H or GFP-CAP-H2 and BMI1A-MYC. Co-IP experiments were conducted using *Nicotiana benthamiana* leaf transiently expressing GFP-CAP-H or GFP-CAP-H2 and BMI1A-MYC. Asterisk indicate GFP-CAP-H2

To further investigate the mechanisms of BMI1s regulating the chromatin 3D structure, especially CD, we screened by yeast two-hybrid assays to find out the interactors of BMI1s. We chose BMI1A as the bait and found that the condensin component CAP-H interacts with BMI1A, which indicated that condensin complexes might be the potential partners of BMI1s. Considering that there are two types of condensin complexes in *Arabidopsis*, condensin I and condensin II (Fig. S1) (Kalitsis et al. 2017), we verified the interactions between BMI1A and both kleisin proteins (CAP-H in condensin I and CAP-H2 in condensin II) through three additional in vitro experiments. The yeast transformed in both plasmids that contain CAP-H/H2 and BMI1A coding sequence, respectively, can survive on the culture medium lacking histidine, leucine, and tryptophan, which showed that both CAP-H and CAP-H2 interact with BMI1A (Fig. 1A). Meanwhile, we performed bimolecular fluorescence complementation (BiFC) experiments. We fused both CAP-H and CAP-H2 with the C-terminal fragment of yellow fluorescent protein (YFP) and BMI1A with the N-terminal fragment. The fusion proteins were driven by the cauliflower mosaic virus (CaMV) 35S promoter in tobacco. The YFP florescent signals were only detected in the leaves expressing the combination of cYFP-CAP-H/H2 and nYFP-BMI1A, which also showed that CAP-H/H2 and BMI1A have interactions (Fig. 1B). Moreover, co-immunoprecipitation (Co-IP) further confirmed that CAP-H and CAP-H2 interact with BMI1A. When BMI1A-MYC and GFP-CAP-H/H2 were co-expressed in tobacco leaves, BMI1A-MYC can be co-immunoprecipitated by GFP-CAP-H/H2. In contrast, when BMI1A-MYC was expressed alone, BMI1A-MYC could not be pulled down by the GFP-Trap (Fig. 1C). BiFC results showed that BMI1B/C and CAP-H/H2 have interactions (Fig. S2A). Additional Y2H results revealed that the interactions between BMI1B/C and CAP-H/H2 are indirect (Fig. S2B). Taken together, we confirmed that BMI1A interacts with condensin subunits CAP-H and CAP-H2, in vitro, which indicates that BMI1s and condensin complexes may function together in *Arabidopsis*.

### BMI1s and condensin complexes do not co-regulate H2AK121ub and H3K27me3 level

**Fig. 2:**
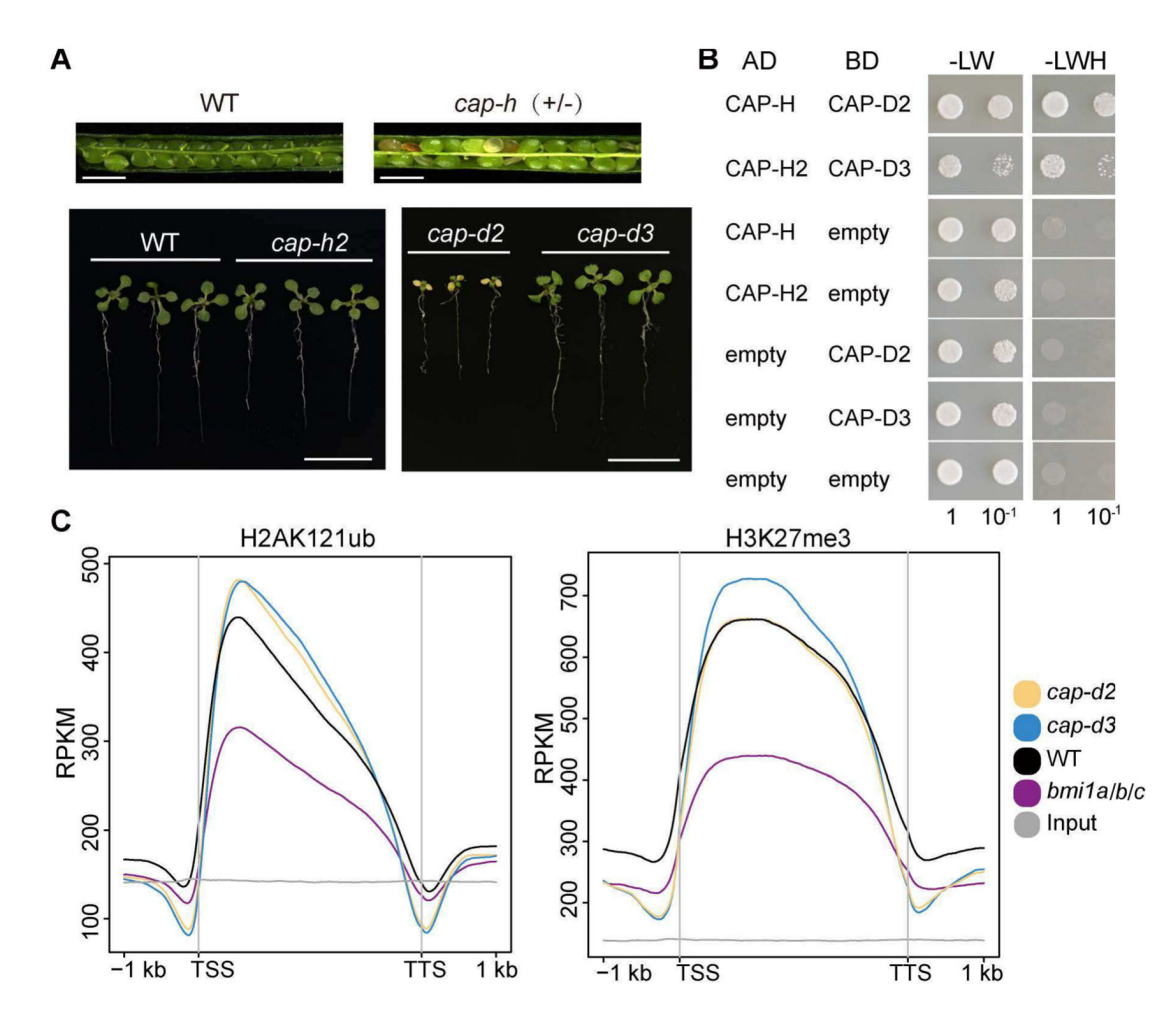
BMI1s and condensin complexes do not co-regulate H2AK121ub and H3K27me3 level. **A** The photos on the top show the siliques of WT plant (left) and *cap-h* mutant (right). Scale bar: 1 mm. Normal morphology of developing seeds was seen in siliques of WT plant. Empty spaces and aborted seeds were seen in silique of *cap-h* mutant. The photos on the bottom show the phenotypes of 10-day-old WT, *cap-h2*, *cap-d2* and *cap-d3* (from left to right) seedlings. Scale bar: 1 cm. **B** Yeast two-hybrid assays show that CAP-H interacts with CAP-D2 and CAP-H2 interacts with CAP-D3. **C** Meta plots show the H2AK121ub levels on H2AK121ub marked genes (left) or H3K27me3 levels on H3K27me3 marked genes (right) in WT plant (black), *bmi1a/b/c* (purple), *cap-d2* (yellow) and *cap-d3* (blue) mutants. Grey line indicates the input signal. 1 kb regions upstream of the transcription start site (TSS) and downstream of the transcription termination site (TTS) are shown

Having confirmed the interaction of condensin I or II and BMI1s in vitro, we wondered whether condensin complexes and BMI1s function together in vivo. Considering that BMI1s have H2A ubiquitin ligase activity and H2AK121ub is essential for BMI1s’ functions, we first checked the histone modification level when disturbing BMI1s, condensin I or condensin II. We used *bmi1a/b/c* mutant to observe the changes brought by losing BMI1s. However, as for disturbing condensin I, the *cap-h* homozygous mutant is embryonic lethal (Fig. 2A). As seen in the model (Fig. S1), despite sharing the core SMC proteins (SMC2A, SMC2B, and SMC4), condensin I and condensin II have different but homologous associated proteins (Kalitsis et al. 2017; Municio et al. 2021). Except for CAP-H and CAP-H2, CAP-D2 and CAP-D3 are also identified as specific components of the condensin I and condensin II, respectively. In plants, the published mass spectrometry results checked the composition of condensin I and II and confirmed that condensin I and II form two distinct complexes to function (Municio et al. 2021). We further performed Y2H and discovered the physical interaction between CAP-H and CAP-D2 or between CAP-H2 and CAP-D3 (Fig. 2B), which showed the close connection between condensin subunit D and H. In addition, *cap-d2* and *cap-d3* have been used to represent the mutants that disturbing condensin I and II, respectively (Wang et al. 2017). Based on the above reasons, we finally chose *cap-d2* and *cap-d3* mutants to represent condensin I and condensin II functional deficient mutants, respectively, for the following experiments and analysis.

We performed the H2AK121ub ChIP-seq experiments for *cap-d2, cap-d3*, *bmi1a/b/c* mutants and WT plant (Fig. S3A). The meta plots showed that the H2AK121ub marked genes in both condensin mutants have slightly higher H2AK121ub levels in comparison to that in the WT plant, whereas the H2AK121ub levels significantly decreased in *bmi1a/b/c* mutant (Fig. 2C). This phenomenon showed that loss of condensin I or II does not influence the enzymatic activity of BMI1s, or the interaction between condensin complexes and BMI1s cannot enhance the enzymatic activity of BMI1s. It has been reported that cohesin, which is another SMC complex, recruits BMI1s and affects H2AK121ub levels to regulate gene expression (Zhang et al. 2023). Our results showed that although condensin complexes can also interact with BMI1s, it does not recruit BMI1s to function, which differs from cohesin condition. Considering that BMI1s indirectly regulate the H3K27me3 levels, we made the same comparison for H3K27me3 (Fig. S3B) (Yang et al. 2024). We observed that the H3K27me3 level on H3K27me3 marked genes also showed no obvious changes in the two condensin mutants, whereas the level of H3K27me3 significantly decreased in the *bmi1a/b/c* mutant (Fig. 2C). Therefore, the interactions between BMI1s and condensin complexes are not for co-regulating the H2AK121ub and H3K27me3 level. Based on these results, we propose that condensin complexes and BMI1s may directly co-regulate other aspects through their interaction with each other.

### Condensin complexes participate in chromatin 3D regulation

**Fig. 3:**
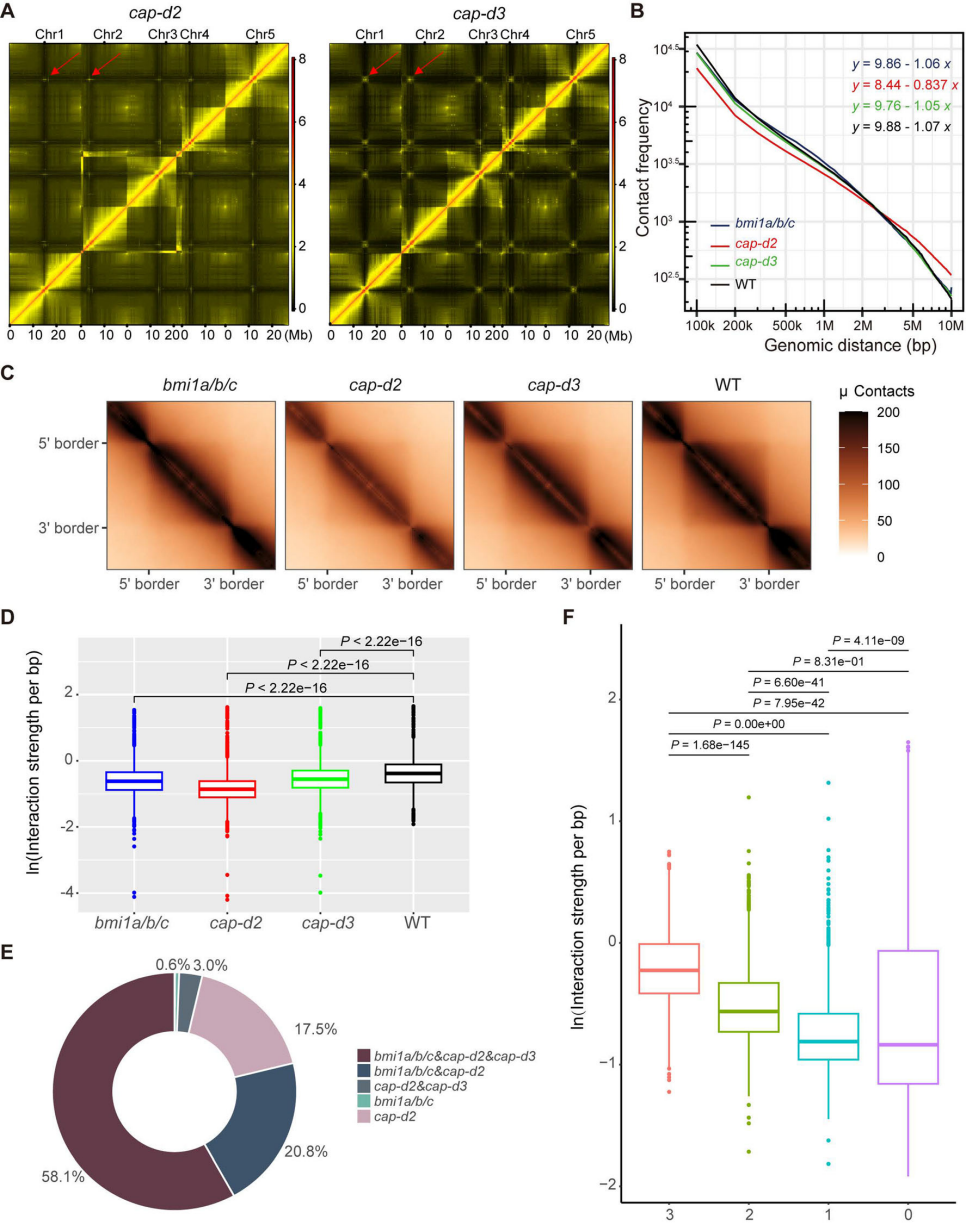
Condensin complexes participate in chromatin 3D regulation and cooperate with BMI1s to maintain CD structure. **A** Hi-C contact matrices of *Arabidopsis* genome in *cap-d2* and *cap-d3* mutants. The five *Arabidopsis* chromosomes are shown. The color code in Hi-C contact matrices represents the frequency of contact. The position indicated by the red arrow represents the interaction between centromeres. **B** Interaction decay exponents (IDEs) of *bmi1a/b/c* (blue), *cap-d2* (red), *cap-d3* (green) mutants and WT (black) plant show the differences in the contact frequency decreasing along the genomic distance from 100 kb to 10 Mb. **C** Aggregate TAD analyses (ATA) show the raw interactions in all CDs in *bmi1a/b/c*, *cap-d2*, *cap-d3* mutants, and WT (from left to right) plant. The color bar indicates the contact intensity. **D** Boxplot shows the interaction strength within CDs in *bmi1a/b/c* (blue), *cap-d2* (red), *cap-d3* (green) mutants and WT (black) plant (from left to right). The median (middle line), upper, and lower quartiles (boxes) are displayed. After conducting the Kruskal–Wallis test, a post hoc analysis using Dunn’s test is performed to determine which groups exhibit significant differences. The *P* values are adjusted using the Bonferroni method. **E** Pie chart shows the percentage of CDs regulated by condensin complexes and/or BMI1s. **F** Boxplot shows the interaction strength within CDs which are regulated by all three complexes (3), any two complexes (2) or any one complex (1), and within the unchanged CDs (0) in the WT plant. After conducting the Kruskal–Wallis test, a post hoc analysis using Dunn’s test is performed to determine which groups exhibit significant differences. The *P* values are adjusted using the Bonferroni method

Considering that condensin complexes were reported to be related to chromatin organization (Yuen and Gerton 2018), we wondered whether condensin complexes may co-regulate chromatin 3D structure with BMI1s. We subsequently performed Hi-C experiments for *cap-d2* and *cap-3* mutants in two biological replicates. High-quality Hi-C read-pairs representative of true interactions were retained after filtering total reads (Fig. S4A). As the two biological replicates demonstrated a high correlation (Fig. S4B), we combined them and obtained 424 and 490 million high-quality read-pairs for *cap-d2* and *cap-d3*, respectively. Since these Hi-C data showed very high sequencing depth, we can analyze other aspects of chromatin 3D structures besides territories and compartments. The matrixes of *cap-d2* and *cap-d3* mutants showed that both condensin I and condensin II indeed influence the chromatin 3D structure (Figs. 3A and S4C) (Yin et al. 2023). Observing the heatmaps of *cap-d2, cap-d3* and previously reported *cap-h2* mutants (Sakamoto et al. 2022), we discovered that the changes of the two condensin II mutants are much more similar. The interaction strength among centromeric regions is significantly increased in both *cap-d3* and *cap-h2* mutants, which is consistent with the previous FISH results that condensin II prevents the abnormal centromere association (Sakamoto et al. 2019; Wallace and Bosco 2013). These results all demonstrated the reliability of our Hi-C data. Since condensin I does not have the role of preventing the abnormal centromere association, we wondered whether BMI1s cooperate with condensin II to regulate the interaction strength among centromeric regions specifically. We observed that, similar to the *cap-d2* mutant, the *bmi1a/b/c* mutant does not display a significant increase in interaction strength among centromeric regions (Yin et al. 2023). Therefore, the function to prevent abnormal centromere association is unique to condensin II. BMI1s do not co-regulate the centromere association with condensin II. BMI1s and condensin complexes all participate in chromatin 3D regulation, and it is possible that they interact to co-regulate chromatin 3D structure, although as different complexes, they have individual roles in specific aspects.

### BMI1s and condensin complexes cooperate to maintain CD structure

Excluding the possibility of co-regulating centromere associations, we obtained the integrated graph of interaction decay exponents (IDEs) for the *cap-d2*, *cap-d3*, *bmi1a/b/c* mutants and WT plants to explore the possible co-regulation of chromatin 3D structure by BMI1s and condensin complexes (Yin et al. 2023). The contact frequency is decayed as the increase of genomic distance in all four plant materials, which reconfirms the reliability of our Hi-C data. More detailed, we discovered that the short-range interactions are all reduced in the *bmi1a/b/c*, *cap-d2* and *cap-d3* mutants and the segment with the genomic distance less than 200 kb of the curves of the three mutants showed a similar trend, although *cap-d2* exhibits the biggest gap in comparison with WT (Fig. 3B). This suggested that BMI1s and condensin complexes may co-regulate short-range interactions. Since the average length of CDs in *Arabidopsis* is less than 50 kb, the interaction within CD is also a form of short-range interactions. In addition, it has been reported that BMI1s/H2AK121ub contribute to maintaining the short-range interactions within CDs (Yin et al. 2023), which leds us to assume that condensin complexes may cooperate with BMI1s to maintain the interactions within CDs. Therefore, we focused on this aspect of CD and further calculated and compared the interaction strength within CDs. Aggregate TAD analyses (ATA) and the box plots showed that the interaction strength within CD is significantly decreased in all three mutants, which indicated that the condensin complexes and BMI1s are all factors functioning to maintain the CDs (Fig. 3C and D). Since the CDs in *Arabidopsis* are divided into four types, we conducted the same comparison for each type of CD, separately. Here, we observed that loss of condensin I, condensin II or BMI1s leads to the impairment of all types of CDs, rather than a specific type of CD. The biggest decline exists in the *cap-d2* mutant and the minimal change belonged to the *cap-d3* mutant, which can also be detected in all four types of CDs (Fig. S5). These results suggested that condensin complexes and BMI1s generally maintain all types of CDs, rather than having strong impact on a specific type of CD.

Next, we calculated the interaction strength within each CD. We defined the CDs whose strength significantly decreased in the *bmi1a/b/c* as BMI1s-regulated CDs. Similarly, we defined the CDs whose strength significantly decreased in the *cap-d2* and *cap-d3* mutants as condensin I- and condensin II-regulated CDs, respectively. We next explored the relationship of the regulated CDs by the three complexes. According to the pie chart, we discovered that the CDs co-regulated by the three complexes account for the vast majority of their respectively regulated CDs (Fig. 3E). This result showed that although the influence degree of BMI1s and condensin complexes on each type of CD may vary, the regulated CDs are basically the same, which indicated that condensin complexes and BMI1s interact with each other to co-regulate CDs. To investigate the characteristics of the co-regulated-CDs, we compared the interaction strength within CDs that are regulated by all three complexes (3), any two complexes (2) or any one complex (1), and within the unchanged CDs (0). We observed that, in the WT plant, the CDs co-regulated by all three complexes have a higher interaction strength than CDs co-regulated by any two complexes. Meanwhile, the interaction strength within CDs co-regulated by any two complexes is significantly higher than that within the CDs regulated by any one complex (Fig. 3F). This phenomenon showed that the roles of condensin complexes and BMI1s for maintaining CDs have an additive effect, which further indicated that condensin complexes and BMI1s work, in concert, to maintain CD structure, in parallel with H2AK121ub.

### BMI1s and condensin complexes co-regulate a set of genes

**Fig. 4:**
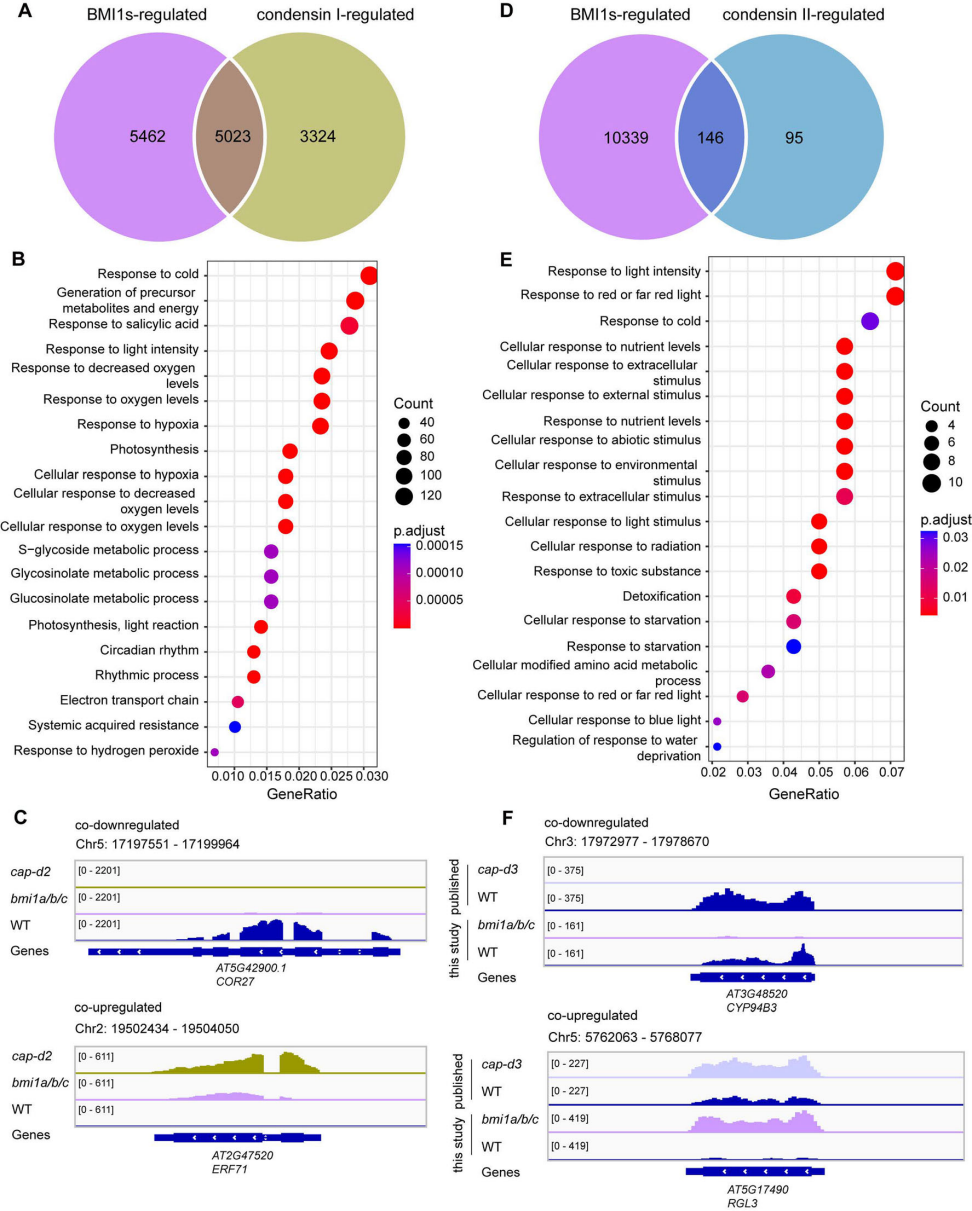
BMI1s and condensin complexes co-regulate a set of genes. **A** Venn diagram shows the overlaps of the differentially expression genes between *bmi1a/b/c* (BMI1s-regulated) and *cap-d2* mutants (condensin I-regulated). **B** GO analysis of common DEGs between *bmi1a/b/c* and *cap-d2* mutants. **C** Expression levels of representative co-downregulated (up) or co-upregulated (down) gene in *cap-d2* and *bmi1a/b/c* mutants. The values are represented by RPKM (reads per kilobase per million mapped reads). **D** Venn diagram shows the overlaps of the DEGs between the *bmi1a/b/c* (BMI1s-regulated) and condensin II mutants (condensin II-regulated). DEGs in *cap-d3* and *cap-h2* mutants were pooled to represent the DEGs when losing condensin II. **E** GO analysis of common DEGs between the *bmi1a/b/c* and condensin II mutants. **F** Expression levels of representative co-downregulated (up) or co-upregulated (down) gene in the *cap-d3* and *bmi1a/b/c* mutants. The values are represented by RPKM

Finally, we quantified the transcripts for WT, *bmi1a/b/c*, and condensin mutant plants to evaluate the consequence of losing condensin I, condensin II, and BMI1s. We performed the RNA-seq experiments for the *bmi1a/b/c* and *cap-d2* mutants, whose quality we checked by a repeatability test (Fig. S6). The differentially expressed genes (DEGs) in *bmi1a/b/c* and *cap-d2* mutants were defined to be BMI1s and condensin I-regulated genes, respectively. We also obtained the published RNA-Seq data for WT, *cap-d3*, and *cap-h2* mutant plants (Municio et al. 2021; Sakamoto et al. 2022; Yang et al. 2022). We pooled the DEGs in the *cap-d3* and *cap-h2* mutants together to represent the genes regulated by condensin II. Venn diagram analysis showed that the differentially expressed genes (DEGs) between the *bmi1a/b/c* and *cap-d2* mutants significantly overlapped (Fig. 4A), which indicated that BMI1s and condensin I co-regulate a set of genes. Genes co-regulated by BMI1s and condensin I enriched in Gene Ontology (GO) terms related to basic biological processes such as photosynthesis, respiration, metabolic process, and environmental response to cold or light (Fig. 4B). Consistently, the disturbance of photosynthesis was related to yellowing of cotyledons in the *cap-d2* or *bmi1a/b/c* mutant (Fig. 2A) (Yang et al. 2013), which further threatens plant survival. For example, the genome browser images of some representative genes are shown. *COLD REGULATED GENE* (*COR27*), which is related to cold response and light, is co-downregulated in the *cap-d2* and *bmi1a/b/c* mutants. Gene associated with response to hypoxia, such as *ETHYLENE RESPONSE FACTOR 71* (*ERF71*), is both upregulated in the two mutants (Fig. 4C). Although loss of CAP-D3 or CAP-H2 leads to less influence on gene transcription, BMI1s and condensin II also co-regulated a portion of genes (Fig. 4D). Similarly, GO analysis showed that the common genes regulated by BMI1s and condensin II are related with various environmental responses (Fig. 4E). As an example, in the *cap-d3* and *bmi1a/b/c* mutants, the wound response gene *CYTOCHROME P450, FAMILY 94, SUBFAMILY B, POLYPEPTIDE 3* (*CYP94B*) is abnormally repressed, whereas *RGA-LIKE PROTEIN 3* (*RGL3*), which is related to dark-induced senescence, is activated (Fig. 4F). Considering that we used the published RNA-Seq data for condensin II mutants, we further extracted the RNA for WT, *cap-d3*, and *bmi1a/b/c* mutant plants to verify the changes in the representative genes. The RT-qPCR results confirmed the RNA-Seq results (Fig. S7). These findings showed that BMI1s co-regulates a set of genes together with condensin complexes. Previous studies have reported that although the specific interactions adjusted in response to cold, the large-scale chromatin conformations were unchanged in *Arabidopsis* (Zhang et al. 2024), we speculated that BMIs and condensin complexes may contribute, cooperatively, to stabilize the CD structures, after cold stimuli, to preferably maintain genome integrity, which may be a different strategy adopted by plants in response to different stresses.

## Discussion

In this study, we identified new global regulators of chromatin 3D structure, condensin complexes, interacting with BMI1s in *Arabidopsis* to cooperatively maintain the interaction strength within the CDs. The co-regulated CDs by these three complexes exhibit higher strength than other CDs, which provides a necessary and appropriate chromatin 3D condition for the normal expression of some genes and may contribute to the maintenance of genomic stability under a stress condition. Moreover, loss of condensin complexes does not disturb H2AK121ub or H3K27me3 levels, genome wide, which shows that the cooperative role of condensin complexes and BMI1s is not dependent on influencing the modification levels, but parallels the function of histone modifications deposited by PcG. This finding supplements mechanism of PRC1 in regulating CDs from a perspective other than modifications.

The chromatin 3D regulatory network is huge and complex. As for chromatin 3D regulatory function of condensin complexes, previous studies focused on the aspects of chromosome territories or compartments (Sakamoto et al. 2022), whereas our high-quality Hi-C data point to CDs. We discovered that condensin complexes are required to maintain the CD structures, which may explain the reports that the euchromatin density in condensin I and II mutants is decrease in the interphase nucleus (Schubert et al. 2013). Furthermore, our results revealed the cooperative roles of condensin complexes and BMI1s in CD regulation. Condensin complexes are well known for their chromatin compaction role in cell division (Gibcus et al. 2018). Maybe in interphase, the capability still exists. The maintenance of some strong CDs may not only need specific histone modifications but also the chromatin compaction factors. BMI1s coordinate both H2AK121ub and condensin complexes simultaneously to regulate CDs. In addition to their joint function for regulating CDs, each complex has multiple functions at different chromatin 3D aspects. On the one hand, we also discovered that condensin II regulates the centromere association independent of condensin I and BMI1s. On the other hand, the IDEs showed that the long-range interaction changes are different in *bmi1a/b/c* and condensin mutants, indicating the presence of distinct regulatory mechanisms and other accessory chromatin 3D regulators. BMI1s can also interact with another SMC complex, cohesin, and be recruited by it, which may contribute to maintaining other chromatin 3D structures (Zhang et al. 2023). In other species, condensin complexes were also discovered to be involved in the formation of territories and compartments or regulating the long-range interactions (Hoencamp et al. 2021; Li et al. 2015; Lioy et al. 2018; Yuen et al. 2017). The research on the function of condensin complexes in interphase is relatively limited in comparison with the deep study of function during cell division. Therefore, there is still a lot worth further exploring for condensin function in *Arabidopsis*, especially in interphase chromatin organization. This research supplements the regulatory mechanism of CD and displays additional chromatin 3D regulatory pathways worth studying.

In *Drosophila*, there are various boundary binding proteins to influence the CD strength (Moretti et al. 2020). We suggest that multiple boundary binding proteins also exist in *Arabidopsis*. PWOs specifically bind to H3K27me3-CD boundary and EMF1 binds to the boundary of all three modification-dominated CDs. PWOs and EMF1 all function to maintain the CDs whose boundary they bind to (Shu et al. 2024; Yang et al. 2024). Condensin complexes may also belong to them, since they tend to locate at domain boundaries in some species (Li et al. 2015; Yuen et al. 2017) and were discovered to maintain CDs in *Arabidopsis*. In addition, as we mentioned in the results, the strength of CDs regulated by all three complexes, by any other two complexes, by any one complex is successively decreased, which implies that similar to *Drosophila*, there are more than one protein function to regulate boundaries and the more the regulatory proteins, the stronger the boundary strength. The strong boundaries are considered to stabilize the interactions within the corresponding domain (Li et al. 2015; Moretti et al. 2020). Moreover, the regulation specificity for different kinds of modification-enriched CDs may be achieved by the cooperation of other assistant factors. Although more experimental evidence is required, our results provide the possibility that condensin complexes are the boundary binding complexes in *Arabidopsis* and suggest the presence of their partners.

Condensin I and II have consistent impact on CDs and it seems that condensin I have a stronger and more general role than condensin II. However, the published data showed that condensin I is hardly present in nuclear in interphase and we also noticed this phenomenon (Fujimoto et al. 2005; Kim et al. 2013). In addition to this study, several previous studies also mentioned that condensin I has function in interphase. It has been reported that condensin I contributes to the maintenance of genomic stability during interphase, since it plays a role in reducing B-induced DNA damage (Sakamoto et al. 2011). Moreover, loss of condensin I subunit CAP-D2 leads to a more significant abnormal activation of transposon than that seen in condensin II mutants (Wang et al. 2017). Therefore, how condensin I works is unclear, which is an interesting and challenging scientific issue. In mammalian cells, some condensin I proteins remain in nucleus in interphase (Schmiesing et al. 2000), which may also be the case in plant cells, yet the reason why a small amount of these proteins has a significant effect is still difficult to elucidate. Considering that condensin I regulates almost all the condensin II-regulated CDs, we guess that the CD regulatory roles of condensin II in interphase need the function of condensin I during mitosis. The maintenance of the rest of condensin I-regulated CDs may need the help of other chromatin 3D regulators. Moreover, condensin I has been reported to compact the transcriptionally activated regions in mitosis, thus enabling the normal cell division (Sutani et al. 2015), which may further influence the interphase chromatin 3D structure. Our results identify a phenomenon that may link interphase and mitosis chromatin structural formation, providing a new research direction.

BMI1s and condensin co-regulate the expression of a portion of genes. In addition to the histone modifications, the accurate chromatin 3D structure cooperatively provides an appropriate environment for gene expression. Multiple regulatory pathways are beneficial for making flexible changes when encountering environmental stimuli or other conditions. However, the adjustment also needs to be moderate and ensure the survival of plants. When subjected to various stress, organisms need some factors to maintain the overall framework of the 3D genome. We proposed that BMI1s and condensin complexes may contribute to the genome integrity under stress. Moreover, BMI1s are involved in two regulatory pathways, simultaneously, which suggests that these multiple regulatory pathways are not independent and may be mutually reinforcing. Therefore, understanding the regulatory mechanism of chromatin 3D structure is beneficial for further research on gene expression regulation.

## Materials and methods

### Plant materials and growth conditions

All ecotypes of *Arabidopsis thaliana* plants used in this study are Columbia-0 (Col-0). The condensin mutants *cap-d2* (Salk_077796) (Wang et al. 2017), *cap-d3* (Salk_094776) (Wang et al. 2017), *cap-h* (Salk_072400) and *cap-h2* (Salk_204300) were obtained from ABRC stock center at Ohio State University. The seeds of *bmi1a/b/c* mutant were provided by Myriam Calonje (Yin et al. 2023). Genotypes of mutants were identified by PCR (for oligonucleotide sequences, see Key Resource Table).

Seeds were sterilized with 10% NaClO Solution and incubated at 4 °C in the dark for 2 days to stratify germination. *Arabidopsis thaliana* plants were grown on MS medium (containing 1% [w/v] sucrose and 0.7% [w/v] agar) in a Percival growth chamber for about 10 days and then moved to soil in greenhouse both at 22 °C under long-day (16-h-light/8-h-dark) conditions. To show the phenotype, photos for 10-day-old seedlings and siliques of 1.5-month-old plants were taken. For RNA-seq, ChIP-seq, and Hi-C experiments, materials from 10-day-old seedlings were collected.

### Plasmid construction

All primers used in this study are listed in Key Resource Table. For the transient transcription assays, the entry vectors containing full-length BMI1A/B/C coding sequence were obtained from our laboratory universal plasmid library. The full-length CAP-H and CAP-H2 coding sequence were amplified and inserted into *Not* I/*Sgs* I sites of the entry vector pDONOR321 using in-fusion (ClonExpress II One-Step Cloning Kit, Vazyme C112-01) methods. The target fragments were subsequently subcloned into the destination vector using gateway methods. pDEST-GBDT7-BMI1A, pDEST-GADT7-CAP-H, and pDEST-GADT7-CAP-H2 were constructed for yeast two-hybrid. For transient transcription in *Nicotiana benthamiana*, pCL112-BMI1A, pCL112-BMI1B, pCL112-BMI1C, pCL113-CAP-H, and pCL113-CAP-H2 were constructed to perform bimolecular fluorescence complementation (BiFC) experiments, pENSG-GFP-CAP-H and pENSG-GFP-CAP-H2 were constructed to perform co-immunoprecipitation (Co-IP) experiments. The BMI1A coding sequence without stop codon was cloned into the *Sal* I/*Spe* I sites of pSUPER1300 fused with 6 × MYC using in-fusion methods for Co-IP.

### Yeast two-hybrid (Y2H)

Bait and prey plasmids, or blank plasmids pGADT7 and pGBKT7 were co-transformed into yeast competent cells (strain Y2H Gold). Complemented SD medium minus Trp (tryptophan) and Leu (leucine) were used for selecting the transformed clone, and complemented SD medium minus Trp, Leu, and His (histidine) were used for checking the interaction. The results were tested after 3–5 days of growth at 28 °C.

### Bimolecular fluorescence complementation (BiFC)

pSOUP colonies containing corresponding constructs and P19 were grown in liquid LB medium with corresponding antibiotic at 28 °C for 16 h. Centrifuge to collect the agrobacterium and resuspend in infiltration buffer (1 mM MgCl_2_, 1 mM MES pH 5.7, 150 mM acetosyringone). The OD_600_ value was measured and adjusted to 3 with infiltration buffer. Equal volume of two kinds of agrobacterium containing corresponding constructs and P19 were mixed. The third or the fourth leaves of *Nicotiana benthamiana* were infiltrated by the syringe without needle and the infiltrated region were labeled. After growing at long-day condition for 2–3 days, a Zeiss LSM 900 Airyscan 2 system and a 20 × objective was used to detect fluorescent signals and take images. Images were processed using the ZEN Microscopy Software.

### Co-immunoprecipitation (Co-IP)

pSOUP or pMP90RK colonies containing corresponding constructs and P19 were grown in liquid LB medium with corresponding antibiotic at 28 °C for 16 h. Tobacco infiltration was the same as mentioned above (BiFC). For one reaction, 0.3 g *Nicotiana benthamiana* leaves were harvested, frozen, and homogenized in liquid nitrogen. The powder was fast transferred to a new tube and resuspended in EWB buffer (50 mM Tris–HCl pH 7.5, 150 mM NaCl, 10% [v/v] glycerol, 2 mM EDTA, 5 mM DTT, 0.1% [v/v] Triton X-100, 0.1% [v/v] protease inhibitor cocktail). After rotating at 4 °C for 20 min, the supernatant was collected and transferred to a new tube through centrifuging at 4 °C at the maximum speed for 15 min. 50 μL of the supernatant was taken out as input before adding the GFP-Trap (ChromoTek, gta-20). The input was stored at 4 °C. The rest supernatant incubated with GFP-Trap beads for 2 h at 4 °C while rotating on a bohemian wheel. The beads were washed for three times using EWB buffer and the proteins were denatured by adding 2 × SDS loading and heated at 95 °C for 10 min. The input was also needed to be denatured. The eluted proteins were detected by western-blot using anti-GFP (EASYBIO, 1:2000 dilution) and anti-MYC (SIGMA, 1:2000 dilution) antibodies to determine the interaction.

### RNA extraction and library preparation

Total RNA was extracted using an E.Z.N.A. Plant RNA Kit (Omega, R6827-01) from 10-day-old seedlings under long-day conditions. About 0.1 g 10-day-old seedlings were used per biological duplication, with three or four biological replicates collected for each sample. RNA-seq libraries were prepared using a VAHTS Universal V8 RNA-seq Library Prep Kit for Illumina (Vazyme, NR605). Briefly, mRNA was purified using VAHTS mRNA Capture Beads (Vazyme, N401) from 3 μg total RNA and fragmented in Frag/Prime Buffer at 85 °C for 6 min. Subsequently, double-stranded cDNA was synthesized, adapters were ligated, and PCR amplification was performed for ten cycles. Libraries were sequenced according to Novaseq 6000 guidelines (Illumina). For quantitative reverse-transcription PCR (RT-qPCR), cDNA was synthetized from the RNA using a One-Step gDNA Removal and cDNA Synthesis SuperMix Kit (TransGen, AE311). RT‐qPCR was performed using the Taq Pro Universal SYBR qPCR Master Mix (Vazyme, Q712) in a Agilent AriaMx Real-time PCR System.

### In situ Hi-C and library preparation

In situ Hi-C was performed as previously described (Yang et al. 2022). Briefly, about 2 g 10-day-old seedlings were used per biological duplication, with two biological replicates collected for each sample. The seedlings were fixed in 1% formaldehyde in MC buffer (10 mM KH_2_PO_4_ pH 7.0, 50 mM NaCl, 0.1 M sucrose) under a vacuum at room temperature, twice for 10 min each. After fixation, seedlings were subjected to a 5 min vacuum treatment with 0.1 M glycine. The fixed seedlings were homogenized in liquid nitrogen for around 20 min, resuspended in nuclei isolation buffer (20 mM HEPES pH 8.0, 250 mM sucrose, 1 mM MgCl_2_, 5 mM KCl, 40% [v/v] glycerol, 0.25% [v/v] Triton X-100, 0.1 mM PMSF, 0.1% [v/v] ß-mercaptoethanol, 0.1% [v/v] protease inhibitor cocktail) before filtered through two layers of miracloth (Merck Millipore). The nuclei were washed twice using nuclei isolation buffer, and 10–20 million nuclei were obtained to continue. Following resuspension in 0.5% (w/v) SDS, nuclei were denatured at 62 °C for 5 min and then treated with 10% [v/v] Triton X-100 for 15 min before digested overnight with 50 U *Dpn* II at 37 °C. The next day, using Klenow Fragment (Thermo Scientific, EP0052), biotin-14-dCTP (Invitrogen, 19,518–018) was incorporated to the digested DNA, which was then blunt-ended. After ligation using T4 DNA ligase, DNA was isolated by phenol–chloroform extraction followed by ethanol precipitation and then sheared by sonication (30 s ON, 90 s OFF, at low intensity, the sonicated DNA size should be mainly distributed in 200–600 bp) using a Bioruptor^®^ Pico (Diagenode). Sheared DNA was size-selected (200–600 bp) using VAHTS^®^ DNA Clean Beads (N411-01) and biotin-14-dCTP labeled DNA was subsequently captured using Dynabeads™ MyOneTM Streptavidin C1 beads (Invitrogen, 65,001). The library was prepared using a VAHTS^®^ Universal Pro DNA Library Prep Kit for MGI (Vazyme, NDM608). Briefly, on-bead end-repair and adapter ligation were performed after biotin enrichment. The beads were washed before re-suspending in 10 mM Tris–HCl buffer (pH 8.0), and DNA was detached from the beads by incubating at 98 °C for 10 min. Ten cycles of PCR were performed to amplify the library molecules. The products were purified using VAHTS® DNA Clean Beads (Vazyme, N411-01). Libraries were sequenced using MGI DNBSEQ T7 platform by generating 2 × 150 bp paired-end reads.

### Chromatin immunoprecipitation and library preparation

ChIP experiments were performed as previously described with some modifications (Yang et al. 2022). Briefly, 1.0 g 10-day-old seedlings grown under long-day conditions were harvested and fixed with 1% formaldehyde in MC buffer (described above) for one biological duplication, with two biological replicates collected for each sample. Seedlings were treated with 0.1 M glycine for 5 min in a vacuum to quench the reaction. The fixed seedlings were homogenized in liquid nitrogen and resuspended in nuclei isolation buffer (50 mM HEPES pH 7.4, 5 mM MgCl_2_, 25 mM NaCl, 5% [w/v] sucrose, 30% [v/v] glycerol, 0.25% [v/v] Triton X-100, 0.1 mM PMSF, 0.1% [v/v] ß-mercaptoethanol, 0.1% [v/v] protease inhibitor cocktail) before filtered through two layers of miracloth. The nuclei were washed twice using wash buffer (16.67 mM HEPES pH 7.4, 6.67 mM MgCl_2_, 33.33 mM NaCl, 13.33% [w/v] sucrose, 13.33% [v/v] glycerol, 0.25% [v/v] Triton X-100, 0.1 mM PMSF, 0.1% [v/v] ß-mercaptoethanol, 0.1% [v/v] protease inhibitor cocktail) and then resuspend in TE-SDS buffer (10 mM Tris–HCl pH 7.4, 1 mM EDTA, 0.25% [w/v] SDS) for sonication. Total chromatin was fragmented to sizes below 500 bp by sonication using a Bioruptor^®^ Pico (Diagenode) (30 s ON, 30 s OFF, at high intensity, 8 min each for about 24 cycles totally). The supernatant containing fragmented DNA was collected through centrifuging and was transferred to a new tube before adding 1.5 times volumes of IP dilution buffer (80 mM Tris–HCl pH 7.4, 230 mM NaCl, 1.7% [v/v] NP40, 0.17% [w/v] DOC), subsequently immunoprecipitated with anti-H2AK119ub (Cell Signaling Technology, 8240) or anti-H3K27me3 (Millipore, 07–449) antibodies overnight. rProtein A Sepharose Beads (Cytiva, 17–19-7901) were balanced using RIPA buffer (0.6 × IP dilution buffer, 0.1% [w/v] SDS) prior to being used to capture H2AK119ub/H3K27me3-associated DNA. Finishing rotating 2 h, DNA coupled beads were washed using RIPA buffer for five times, and the DNA fragments were eluted in the glycine elution buffer (0.1 M glycine, 0.5 M NaCl, 0.05% Tween-20). De-crosslink at 65 °C overnight after adding 1 M Tris–HCl (pH 9.7) and 10% (w/v) SDS. DNA was isolated by phenol–chloroform extraction followed by ethanol precipitation. Two biologically independent DNA libraries were constructed using the NuGEN Ovation^®^ Ultralow V2 DNA-Seq Library Preparation Kit (TECAN, 0344NB) and sequenced using a Hiseq-Xten PE150 platform.

### RNA-seq data analysis

Fastp (Chen et al. 2018) was used to filter the raw sequencing data and Hisat2 (Kim et al. 2019; Pertea et al. 2016) was used to map the reads with gaps to the TAIR 10 reference genome. After filtering, indexing, and converting the format using SAMtools (Danecek et al. 2021), we employed StringTie (Pertea et al. 2016) for transcript quantitation and extracted the read counts. DEseq2 (Love et al. 2014) was used to define differential expression genes (*q* < 0.05 and |log2(fold change) |> 1). Gene ontology (GO) was analyzed and plotted using clusterProfiler (Yu et al. 2012).

### Hi-C data analysis

Fastp (Chen et al. 2018) was used to filter the raw sequencing reads and the HiC-Pro pipeline (Servant et al. 2015) with default parameters was used to map TAIR10 genome and extract valid pairs. HiCdatR (Schmid et al. 2015) was used to compare two Hi-C replicates. Once both replicates were found to be highly correlated, the raw data were merged to obtain higher coverage. Downstream analysis mainly relied on HiCExplorer (Ramírez et al. 2018). hicNormalize with “smallest” and hicCorrectMatirx with “Knight-Ruiz Matrix Balancing (KR)” were used to normalize and balance different sample matrices, respectively. hicPlotDistVsCounts was used to summarize the Hi-C counts enrichment at different genomic distances up to the whole chromosomes and obtain the lDE by linear fitting. CD definition was performed based on the insulation score (IS) computed by hicFindTADs and the min3000_max30000_step1000_thres0.05_delta0.01_fdr parameter was used. hicDifferentialTAD was used to identify the CDs regulated by BMIs and condensin complexes, with -p 0.01 -m all. hicInterIntraTAD can summarize the interaction strength in the CDs. After conducting the Kruskal–Wallis test, a post hoc analysis using Dunn’s test is performed to identify significant differences in strength between the indicated CDs. The *P* values are adjusted using the Bonferroni method. GENOVA can aggregate Hi-C matrix around CDs (van der Weide et al. 2021). The interaction matrix was visualized by HiCPlotter (Akdemir and Chin 2015).

### ChIP-seq data analysis

Fastp (Chen et al. 2018) was used to filter the raw sequencing data and Bowtie2 (Langmead and Salzberg 2012) was used to map the reads to the TAIR 10 reference genome. Mapped reads were indexed, cleaned, and format converted using SAMtools (Danecek et al. 2021). Duplications were subsequently removed using PICARD (http://broadinstitute.github.io/picard/) and significant peaks were identified using sicer (Zang et al. 2009) with -g 200 -egf 0.9 -fdr 0.05. Comparison of the peak binding features was performed using R package ChIPpeakAnno (Zhu et al. 2010). The meta-gene plots were produced using Seqplots (Stempor and Ahringer 2016).

### Accession numbers

The Arabidopsis Information Resource (TAIR) accession numbers of genes involved in this research: *BMI1A* (*At2g30580*), *BMI1B* (*At1g06770*), *BMI1C* (*At3g23060*), *SMC2A* (*At5g62410*), *SMC2B* (*At3g47460*), *SMC4* (*At5g48600*), *CAP-D2* (*At3g57060*), *CAP-G* (*At5g37630*), *CAP-H* (*At2g32590*), *CAP-D3* (*At4g15890*), *CAP-G2* (*At1g64960*), *CAP-H2* (*At3g16730*), *COR27* (*At5g42900*), *ERF71* (*At2g47520*), *CYP94B3* (*At3g48520*), *RGL3* (*At5g17490*).

## Supplementary Information

Below is the link to the electronic supplementary material.

Supplementary file1 (DOCX 2934 KB)

Supplementary file2 (XLSX 10 KB)

Supplementary file3 (XLSX 10 KB)

## Data Availability

Further information and requests for resources and reagents should be directed to and will be fulfilled by the lead contact, Yue Zhou (yue_zhou@pku.edu.cn).
