# Supplementary material for: BMI1s interact with condensin complexes to regulate chromatin 3D structure and gene expression in Arabidopsis
Source: aBIOTECH. 2025 Feb 17;6(3):424–40. doi: 10.1007/s42994-025-00202-x (PMC12454742; doi:10.1007/s42994-025-00202-x)
Supplement: Supplementary file 1 — Supplementary file1 (DOCX 2934 KB) [file 42994_2025_202_MOESM1_ESM.docx]

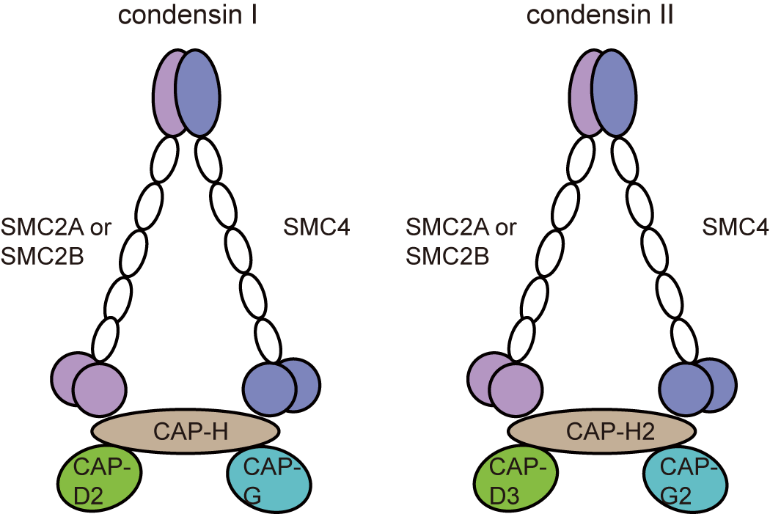


**Fig. S1** Models of Arabidopsis condensin I and condensin II. Related to Fig. 1.

The models show the subunit composition of condensin I and condensin II in Arabidopsis (Kalitsis et al., 2017; Municio et al., 2021).

**
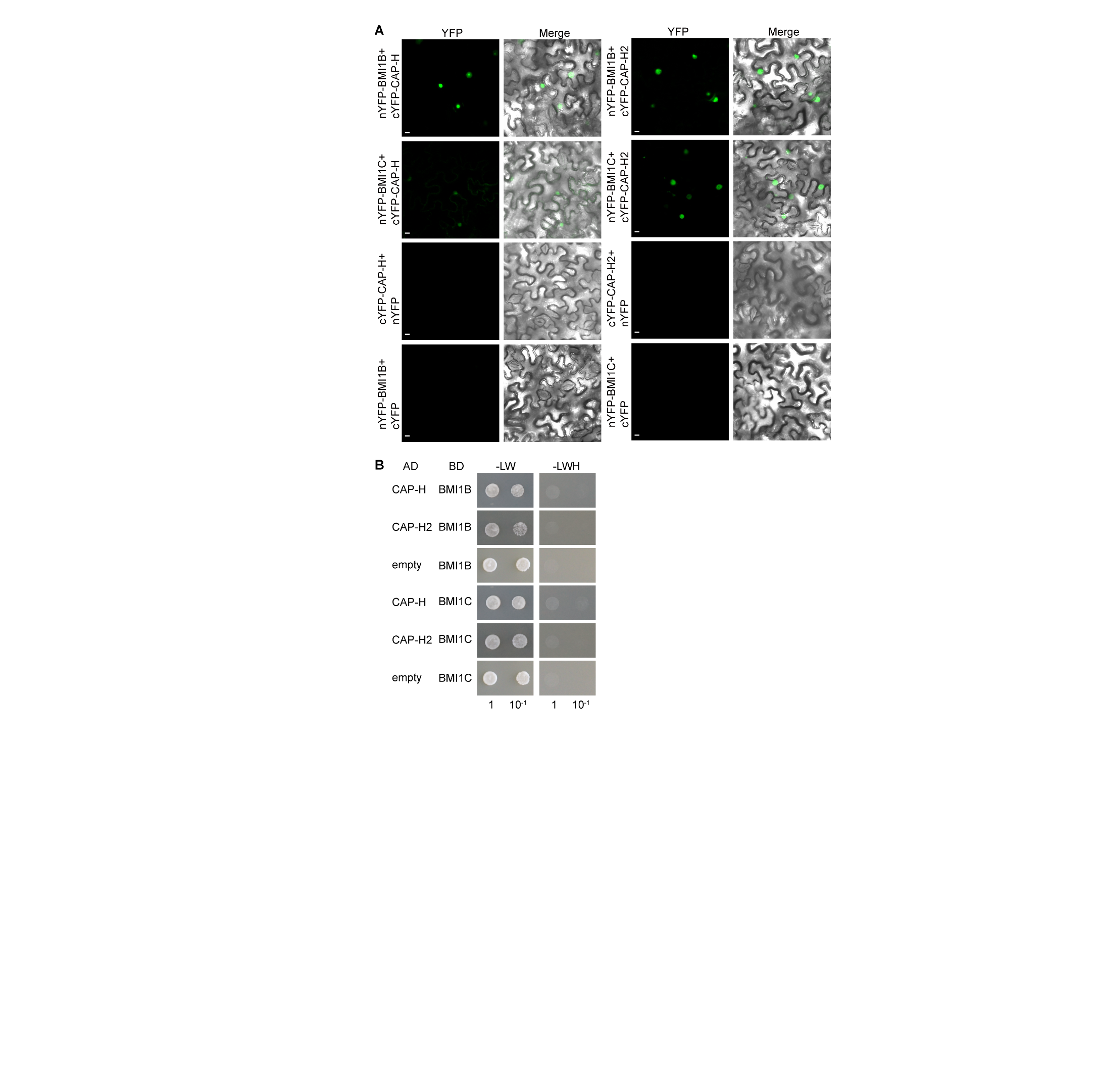
Fig. S2** BMI1B and BMI1C indirectly interact with condensin subunits CAP-H and H2. Related to Fig. 1.

**A** The confocal images of BiFC assay in *Nicotiana benthamiana* leaf show the interaction between cYFP-CAP-H or cYFP-CAP-H2 and nYFP-BMI1B or nYFP-BMI1C. Scale bar: 10 μm.

**B** Yeast two-hybrid assays show that CAP-H/H2 and BMI1B/C do not have direct interactions.


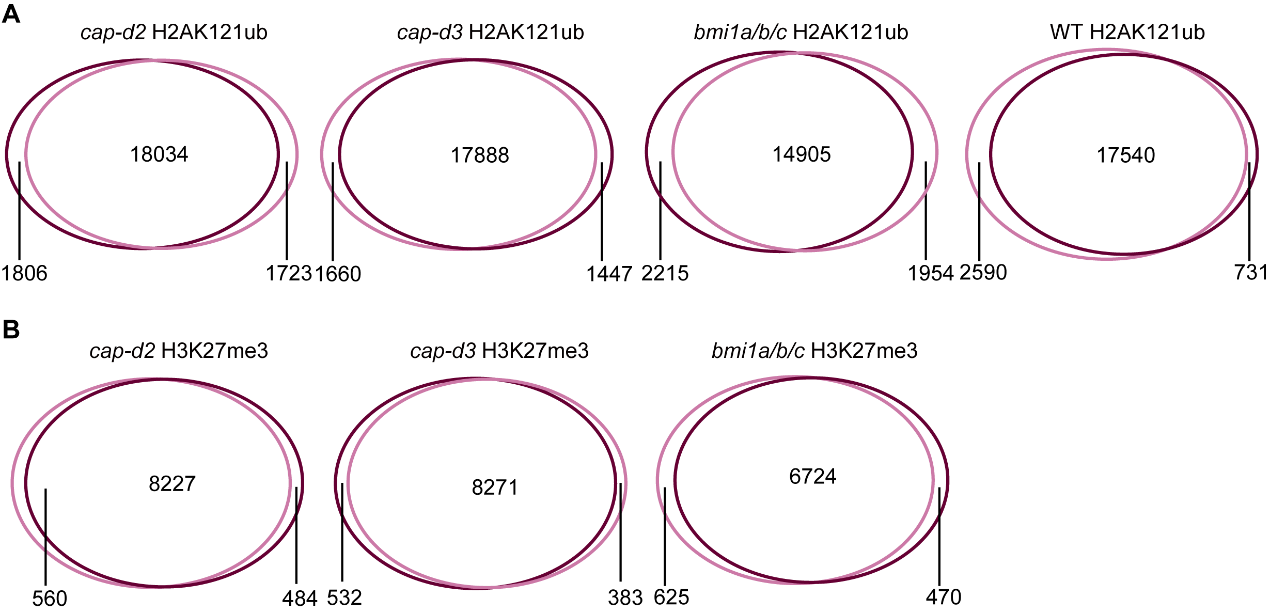


**Fig. S3** ChIP-seq results of *cap-d2,* *cap-d3* and *bmi1a/b/c* mutants and WT plant. Related to Fig. 2.

**A** Venn diagrams show the significant overlap of the peaks between two replicates, which indicates high-quality of ChIP-seq results for H2AK121ub in *cap-d2*, *cap-d3, bmi1a/b/c* mutants and WT plant (from left to right).

**B** Venn diagrams show the significant overlap of the peaks between two replicates, which indicates high-quality of ChIP-seq results for H3K27me3 in *cap-d2* (left), *cap-d3* (middle) and *bmi1a/b/c* (right) mutants.

**
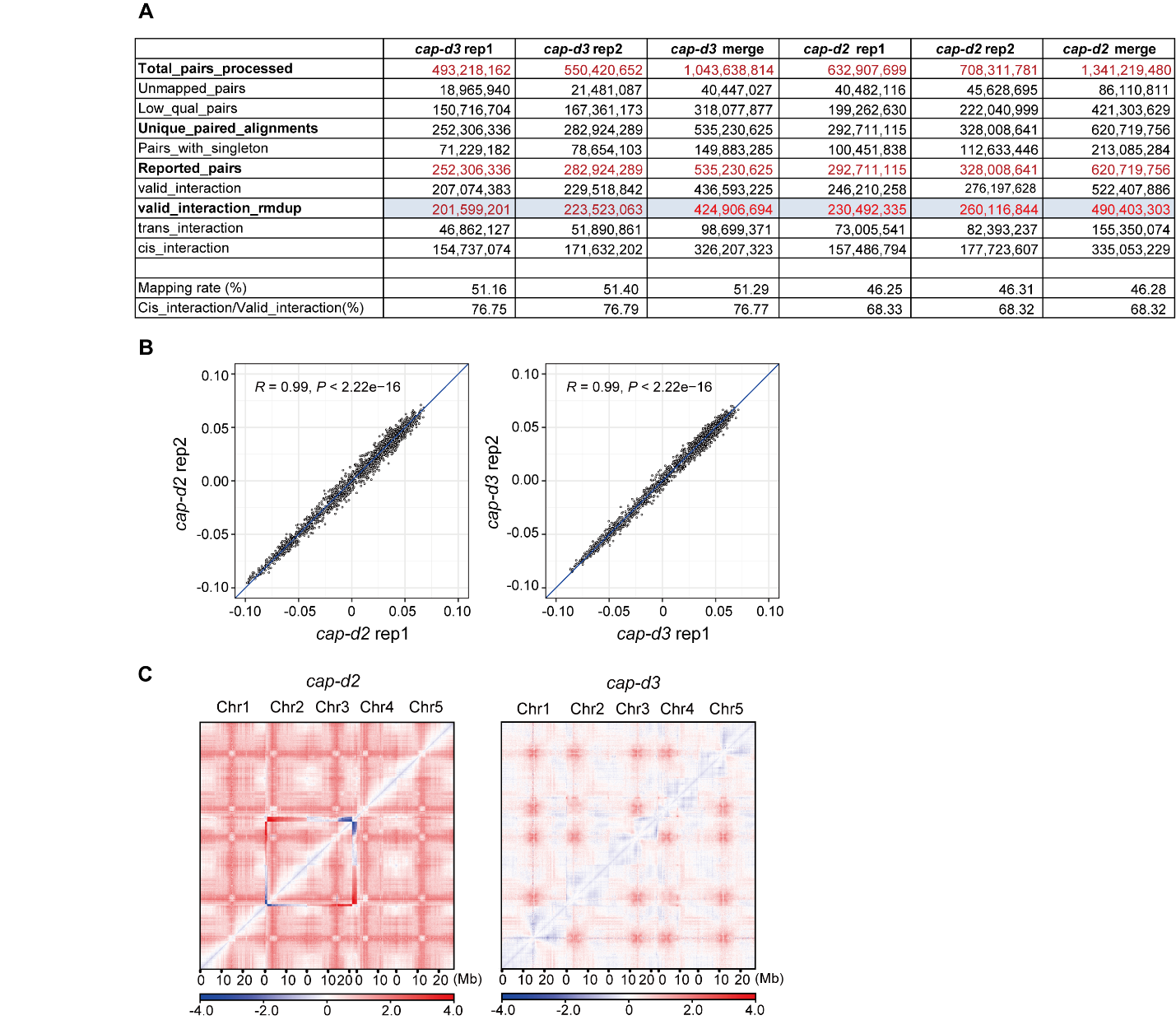
Fig. S4** Hi-C results for *cap-d2* and *cap-d3* mutants. Related to Fig. 3.

**A** Table summarizes the pair and interaction information of the mutant Hi-C data.

**B** Scatter plots of *cap-d2* (left) and *cap-d3* (right) mutants show the Hi-C reproducibility of two biological replicates. Pearson correlation test was used to confirm the repeatability.

**C** Relative interaction heatmaps show the chromatin 3D differences between *cap-d2* (left) and *cap-d3* (right) mutant. Red and blue represent the increased and reduced interaction strength in the mutants, respectively. The color intensity represents the degree of difference.


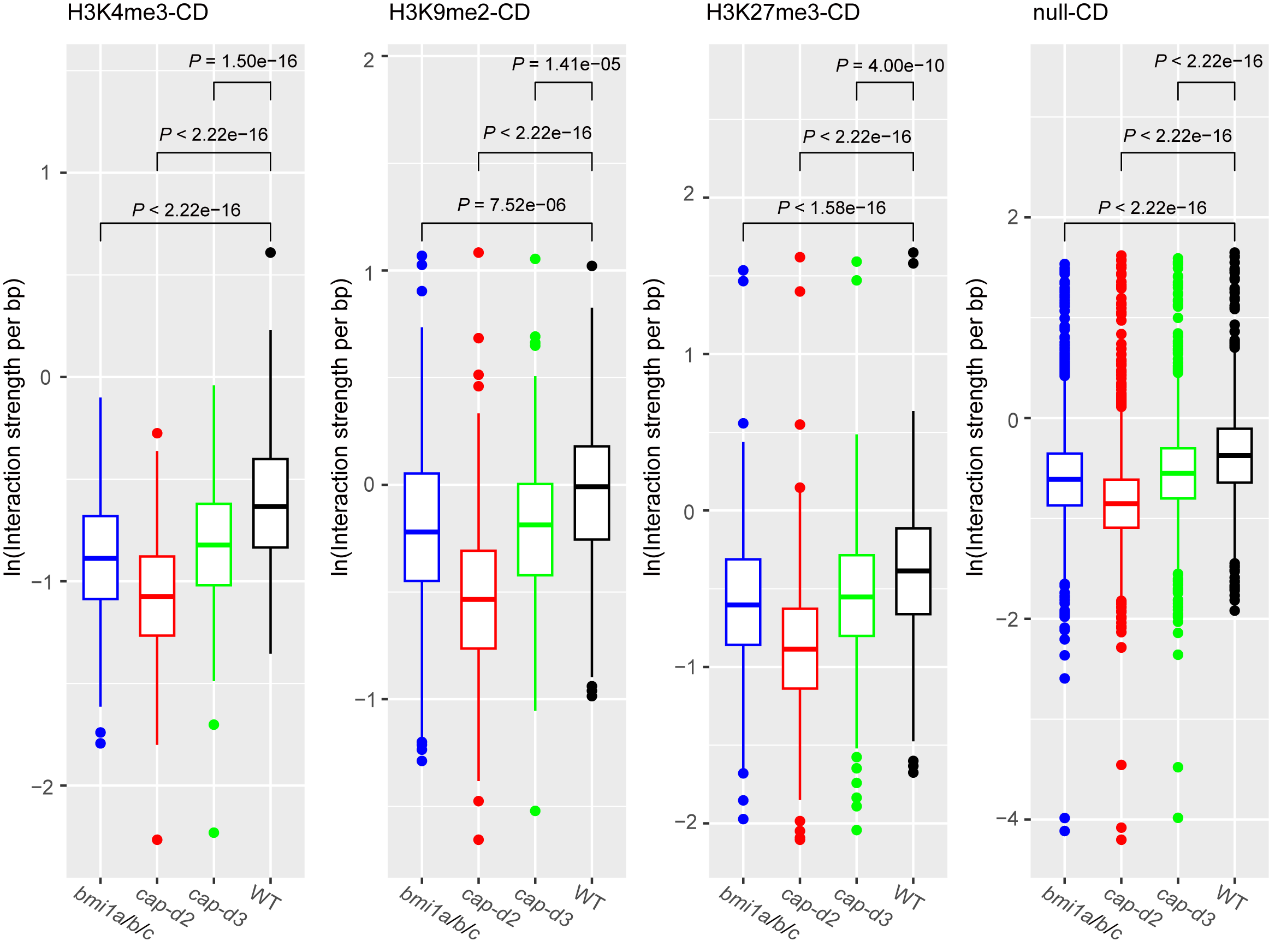


**Fig. S5** BMI1s and condensin complexes maintain all types of CDs. Related to Fig. 3.

Boxplots show the interaction strength within H3K4m3-, H3K9me2-, H3K27me3- and null-CDs (from left to right) in *bmi1a/b/c* (blue), *cap-d2* (red), *cap-d3* (green) mutants and WT (black) plant. The median (middle line), upper and lower quartiles (boxes) are displayed. After conducting the Kruskal-Wallis test, a post-hoc analysis using Dunn's test is performed to determine which groups exhibit significant differences. The *P*-values are adjusted using the Bonferroni method.

**
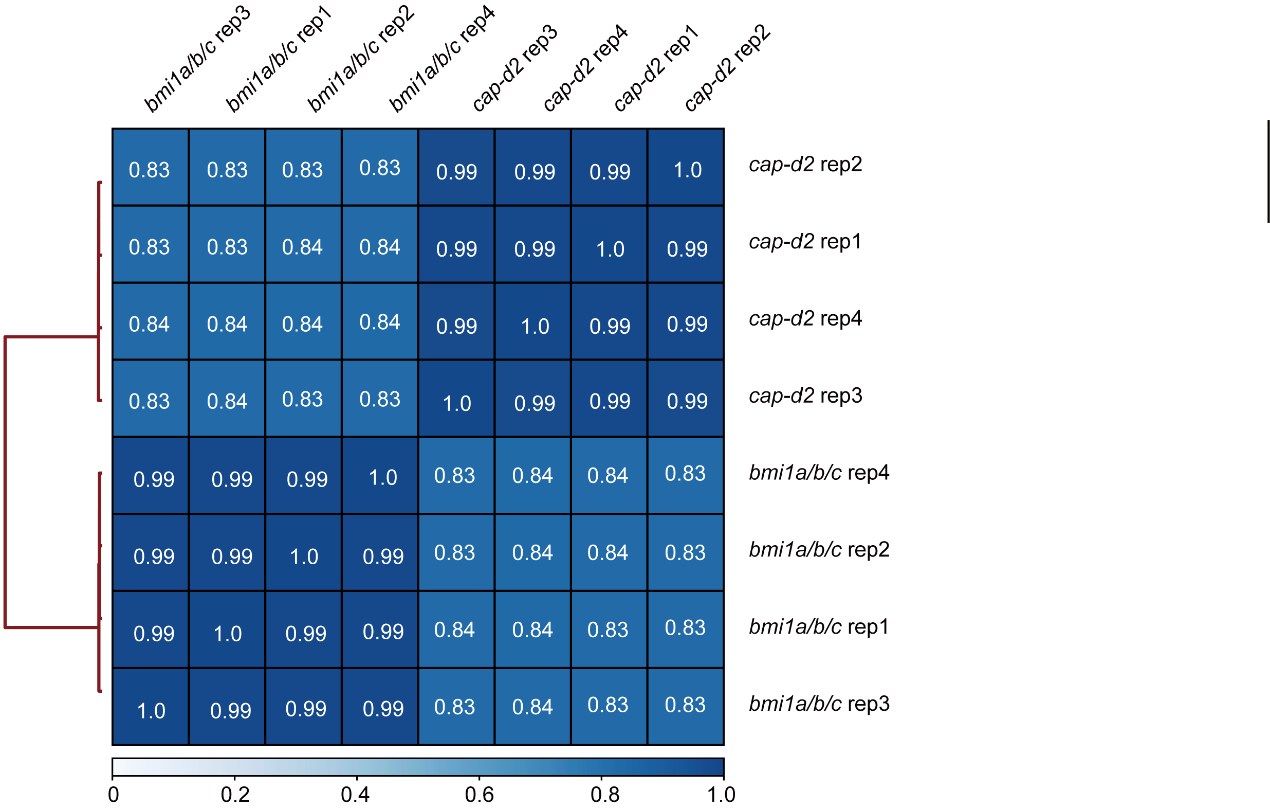
**

**Fig. S6** RNA-Seq results of *cap-d2* and *bmi1a/b/c* mutants. Related to Fig. 4.

Correlation of RNA-seq read counts of *cap-d2* and *bmi1a/b/c* mutants is shown. Repeatability test of RNA-seq is applied by Spearman correlation.

**
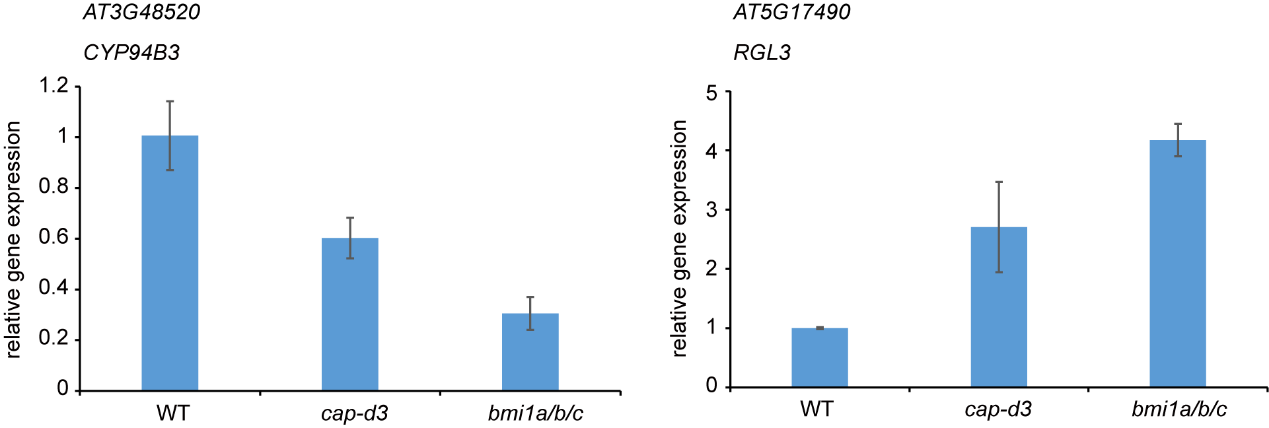
Fig. S7** RT-qPCR results of co-regulated genes by BMI1s and condensin II. Related to Fig. 4.

*CYP94B3* (left) and *RGL3* (right) expression in WT plant, *cap-d3* and *bmi1a/b/c* mutants. *PP2A* was used as the reference gene. Error bars represent standard deviation.
